# Metabolic responses to the occurrence and chemotherapy of pancreatic cancer: biomarker identification and prognosis prediction

**DOI:** 10.1038/s41598-024-56737-4

**Published:** 2024-03-23

**Authors:** Tianhong Teng, Han Shi, Yanying Fan, Pengfei Guo, Jin Zhang, Xinyu Qiu, Jianghua Feng, Heguang Huang

**Affiliations:** 1 Department of General Surgery, Fujian Medical University Union Hospital, Fuzhou, Fujian China; 2 Fuzhou Children Hospital of Fujian Province, Fuzhou, Fujian China; 3 Department of Electronic Science, Fujian Provincial Key Laboratory of Plasma and Magnetic Resonance, Xiamen University, Xiamen, China

**Keywords:** Pancreatic cancer, Serum, Nuclear magnetic resonance, Metabolomics, Chemotherapy, Cancer, Biomarkers

## Abstract

As the most malignant tumor, the prognosis of pancreatic cancer is not ideal even in the small number of patients who can undergo radical surgery. As a highly heterogeneous tumor, chemotherapy resistance is a major factor leading to decreased efficacy and postoperative recurrence of pancreatic cancer. In this study, nuclear magnetic resonance (NMR)-based metabolomics was applied to identify serum metabolic characteristics of pancreatic ductal adenocarcinoma (PDAC) and screen the potential biomarkers for its diagnosis. Metabolic changes of patients with different CA19-9 levels during postoperative chemotherapy were also monitored and compared to identify the differential metabolites that may affect the efficacy of chemotherapy. Finally, 19 potential serum biomarkers were screened to serve the diagnosis of PDAC, and significant metabolic differences between the two CA19-9 stratifications of PDAC were involved in energy metabolism, lipid metabolism, amino acid metabolism, and citric acid metabolism. Enrichment analysis of metabolic pathways revealed six shared pathways by PDAC and chemotherapy such as alanine, aspartate and glutamate metabolism, arginine biosynthesis, glutamine and glutamate metabolism, citrate cycle, pyruvate metabolism, and glycogolysis/gluconeogeneis. The similarity between the metabolic characteristics of PDAC and the metabolic responses to chemotherapy provided a reference for clinical prediction of benefits of postoperative chemotherapy in PDAC patients.

## Introduction

Pancreatic cancer is one of the worst prognosis malignant tumors with a 5-year survival rate of less than 13%^1^. According to statistics, 90% of pancreatic cancer is pancreatic ductal adenocarcinoma (PDAC). Special anatomical location and atypical clinical symptoms make the early diagnosis of pancreatic cancer difficult. In clinical practice, only 15–20% of patients are eligible for radical surgery at the initial diagnosis^2^. In recent years, with the development of systems biology, it has been realized that pancreatic cancer is a highly heterogeneous tumor^3^ and patients are often accompanied by abnormal gene and metabolic changes^4–6^.

For resectable pancreatic cancer, radical surgery is the main treatment strategy, but even with adjuvant chemotherapy, the overall prognosis is not ideal with a 5-year survival rate of only 20–25%. Nevertheless, postoperative chemotherapy has been proven to be effective in delaying tumor recurrence and improving survival. Therefore, adjuvant chemotherapy is recommended for all postoperative patients with pancreatic cancer. Chemotherapy resistance is the main factor leading to decreased efficacy and postoperative recurrence. The mechanism of drug resistance involves multiple genes and multiple signaling pathways. As the downstream events of genes and signaling pathways, the serum metabolites will also change with the emergence of drug resistance.

Metabolomics can analyze metabolites in tissues or body fluids and find out the relationship between metabolites and disease or biological status. Compared with other omics, metabolomics has an “amplification effect”, which can amplify the changes in genes or proteins level, thus reflecting the pathophysiological changes more sensitively. In addition, the cost of detection is low, which has a broad promotion and application prospect in the early diagnosis and monitoring of diseases. Wang et al.^7^ used non-targeted metabolomics to analyze the serum metabolites of patients with newly diagnosed pancreatic cancer. Through screening, it was found that the metabolite combination of aspartate, alanine, androstenediol monosulfate, and glycylvaline could obtain a higher area under the receiver operating characteristic curve (AUC) than Carbohydrate antigen 19-9 (CA19-9) for the early diagnosis of pancreatic cancer patients. Patel et al.^8^ included 354 patients with lung adenocarcinoma undergoing chemotherapy for metabolomics analysis. The biomarker model established by seven metabolites was able to predict the efficacy and survival outcome of chemotherapy before treatment. Phua et al.^9^ found that lactate levels were different among patients with different survival periods through tumor tissues of 25 patients who received preoperative gemcitabine chemotherapy. Some scholars have analyzed the metabolic fingerprint of parental and nab-paclitaxel resistant PDAC cell lines and found that the reduction of aspartate in the nab-paclitaxel resistant cell lines helps to increase the supply of pyrimidine in cells, thus making it easy to acquire resistance to chemotherapy drugs^10^.

CA19-9 is a sialylated Lewis blood group antigen with a normal value of less than 37 U/mL. Since its discovery in 1979, CA19-9 has been reported as the most sensitive tumor marker for pancreatic cancer, playing an important role in the diagnosis and prognosis evaluation of pancreatic cancer^11^. In this study, ^1^H NMR method was used to perform non-targeted metabolomics analysis on the serum of pancreatic cancer patients and healthy subjects to screen out the differential metabolites with potential diagnostic value. Then, the serum of patients with pancreatic cancer undergoing postoperative chemotherapy were analyzed and compared with the changes in CA19-9 level during chemotherapy to identify the metabolic markers affecting the efficacy of postoperative chemotherapy and the involved metabolic pathways during chemotherapy.

## Materials and methods

### Study design and participants

Twenty-nine patients with newly diagnosed PDAC and 30 healthy subjects with similar age and in same gender with the patients were recruited from Fujian Medical University Union Hospital from January 2020 to June 2021. All patients were 18–75 years old, and their pathological stages were determined by surgery or/and imaging examination. All participants were excluded from other endocrine and metabolic diseases such as hyperthyroidism and diabetes, and the demographic data of the patients and the healthy subjects (control group) were shown in Table 1.

**Table 1:** Demographic data of included subjects.

		PDAC group	Control group	Statistics^a^	P value^a^	Normal group	Elevated group	P value^b^
Cases		29	30	–	–	10	14	–
BMI (kg/m^2^)^c^		21.34 ± 1.8	22.19 ± 2.0	t = − 1.717	9.1 × 10^–2^	21.92 ± 1.9	21.22 ± 1.7	3.5 × 10^–1^
Gender (male/female)		17/12	16/14	χ^2^ = 0.167	6.8 × 10^–1^	7/3	8/6	6.7 × 10^–1^
Age (year)		58.83 ± 9.2	56.74 ± 13.0	t = 0.712	4.8 × 10^–1^	61.10 ± 7.8	54.64 ± 8.2	6.4 × 10^–2^
Tumor location (head/body and tail of the pancreas)		19/10	–	–	–	7/3	9/5	1.0 × 10^0^
TNM stage	I	3	–	–	–	0	0	1.0 × 10^0^
	II	18				8	10	
	III	6				2	4	
	IV	2				0	0	

T-test is used to compare the quantitative data between groups. Chi-square test and Fisher’s exact test are used to compare Enumeration data between groups. P < 0.05 indicated statistically significant.

^a^The date of the PDAC patients and the healthy subjects.

^b^The date between the groups with different CA19-9 levels.

^c^Body mass index.

Among 29 patients, 24 patients with stage II or III according to tumor-node-metastasis (TNM) classification system underwent radical surgery^12^. The postoperative chemotherapy regimen was nab-paclitaxel plus gemcitabine. During the chemotherapy, CA19-9 levels were periodically reviewed monthly, and the patients whose CA19-9 levels were consistently higher than 37 U/mL within 3 months during the chemotherapy were assigned as “CA19-9 elevated group” (elevated group), while those with stable CA19-9 at normal level were assigned as “CA19-9 normal group” (normal group). All of the samples from the elevated and normal groups were included in the subsequent analysis.

#### Inclusion criteria of PDAC patients

(1) Age 18–75 years old; (2) first visit, pancreatic tumor diagnosis; (3) preoperative blood test indicated high CA19-9 level (> 37 U/mL), (4) resectable pancreatic cancer was evaluated and radical surgery was performed; (5) post-surgery pathology confirmed PDAC; (6) patients agreed to participate in the study by signing informed consent.

#### Exclusion criteria of PDAC patients

(1) Age below 18 or above 75; (2) co-infection; (3) prior diagnosis or treatment for pancreatic cancer; (4) history of other malignancies; (5) elevated blood bilirubin on admission; (6) inability of patients to comprehend study conditions and objectives.

### Sample collection and preparation

Blood was collected in the morning of the next day after admission after all subjects fasted for more than 8 h. According to a standard clinical procedure, 5 mL of peripheral blood was collected and placed at 4 ℃ for 2 h. The serum was separated and transferred into EP tubes after centrifugation at 3000×*g* for 10 min, and then stored at − 80 °C until analysis.

The serum samples were melted at 4 °C and 400 μL of serum were mixed with 200 μL of 90 mM deuterated phosphate buffer solution (pH 7.4) in an EP tube. After being centrifuged at 10,000×*g* for 10 min at 4 °C, 550 μL of supernatant were transferred into a 5-mm NMR tube (ST500, NORELL, Inc., Morganton, North Carolina, USA) for ^1^H-NMR detection.

### NMR spectral acquisition and preprocessing

^1^H NMR spectra of the samples were obtained using a 600 MHz (Megahertz) Bruker Avance III NMR spectrometer (Bruker Corporation, Kalsruhe, Germany) with a standard water-suppressed CPMG (Carr-Purcell-Meiboom-Gill) pulse sequence at 298 K. The parameters were as follows: spectral width 17 kHz (Kilohertz), relaxation delay time 4.0 s, acquisition time 1.93 s, and data acquisition points 32 K. MestReNova (version: 9.0, Mestrelan Research, Santiago de Compostella, Spain) was used to perform Fourier transforms, phase adjustments, baseline corrections, and other processing on ^1^H NMR spectra. All spectra were multiplied by an exponential weighting function equivalent to a 1 Hz line-broadening to improve the signal-to-noise ratio. Calibrated with lactate doublet at δ1.33, the NMR spectra were integrated at the spectral regions of δ8.498–0.600 with an integration interval of 0.002 ppm. The spectral regions of δ5.724–5.876 and δ4.666–5.080 were removed to eliminate the impacts of residual water and urea. The integrated data were visualized into a data matrix, and then normalized the integral sum of each spectrum to a constant sum (100 in this study) for pattern recognition analysis.

### Statistical analysis

SIMCA (V14.1, Umetrics AB, Umea, Sweden) was used for multivariate statistical analysis of the pre-processed data through pareto scaling. In this study, principal component analysis (PCA) was first used to achieve dimensionality reduction of multidimensional data, and the distribution between different groups was observed in an unsupervised mode. To better extract and maximize metabolome differences between groups, supervised multivariate statistical analyses, including PLS-DA and OPLS-DA, were performed. The permutation test (permutation number = 200) was performed on NMR data to verify potential over-fitting. In addition, correlation coefficient (r) and variable importance in projection (VIP) are obtained from OPLS-DA model. The differential metabolites were screened by referring to r-value, VIP value, and P values from student-t test. VIP > 1 and P < 0.05 were used as screening criteria for differential metabolites, and the critical value of |r| was determined according to the degree of freedom (df) and P value (P = 0.05 in this study).

The statistical data were analyzed by SPSS 29.0 software (SPSS Inc., Chicago, IL, USA). The quantitative data of the normal distribution were expressed as $\overline{{\text{x}} }$ ± s. The comparison between groups adopted the t-test. Enumeration data were expressed in absolute numbers, and the comparison between groups was performed using the Chi-square test and Fisher’s exact test. P < 0.05 indicated statistically significant.

### Metabolic pathway analysis

In order to determine the metabolic disturbance responding to chemotherapy, MetaboAnalyst 5.0 combined with the KEGG database was used for pathway analysis based on the screened differential metabolites^13–15^. The criteria for the disturbed pathway with a sufficient impact on the different groups were “impact” greater than 0.1 and “Hits” not less than 2.

### Ethical approval

The study was approved by the ethical committee of Fujian Medical University Union Hospital (No.: 2020WSJK037). All methods were performed in accordance with the relevant guidelines and regulations. Written informed consent was received from participants before inclusion in this study.

### Informed consent

Informed consent forms were signed by all participating patients.

## Results

### Metabolic characteristics of PDAC patients

To understand the metabolic characteristics of PDAC patients and identify the metabolic differences between the pancreatic cancer patients and healthy subjects, the serum samples of the two groups were analyzed by metabolomics strategy. No significant difference was observed in age, gender and body mass index (BMI) between the PDAC and control groups (P > 0.05) (Table 1).

**Figure 1:**
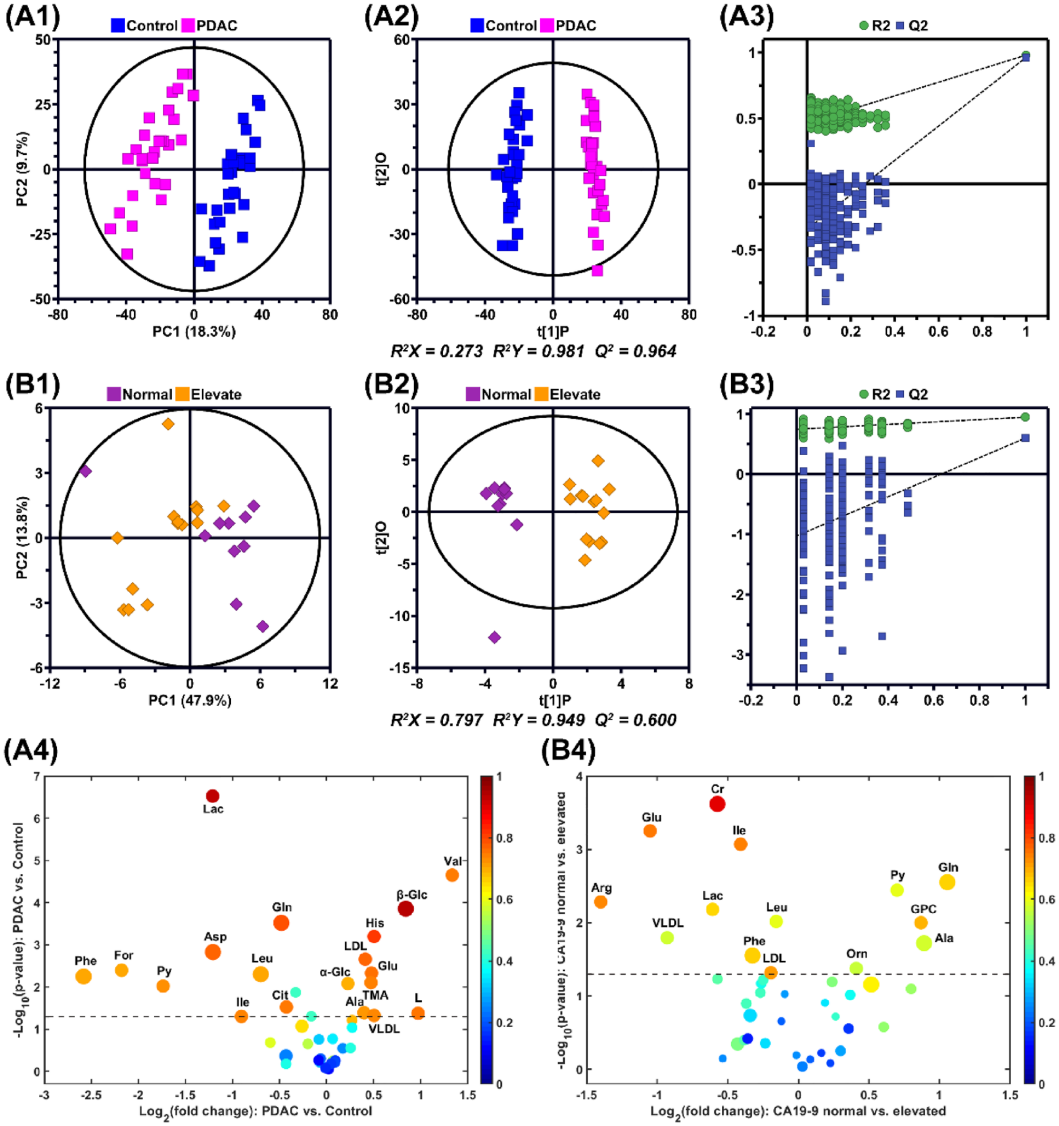
PCA (**A1,B1**), OPLS-DA (**A2,B2**) score plots, the corresponding permutation test (n = 200) (**A3,B3**) derived from the NMR data, and the volcano maps (**A4,B4**) of discriminative metabolites between the PDAC patients and the healthy controls (**A**) and between the CA19-9 elevated and normal groups (**B**). In the volcano maps, each point represents a metabolite. The color of points represents the absolute value of r, and the size of points represents the VIP value. The dash line represents the logarithmic value of P = 0.05.

PCA scores plot of the PDAC patients and controls was shown in Fig. 1A1, and the first two principal components respectively provide 18.3% and 9.7% explanation rates for the model and an obvious separation is shown between the patients and the controls. Supervised pattern recognition method PLS-DA maximized the system variance and eliminated interference information of the NMR data and the results of permutation test indicated no overfitting of the model (Fig. S1A,B). In addition, OPLS-DA was performed to better extract the metabolome differences between the different groups. The results show that there was a significant separation between the PDAC group and the control group, and the R^2^X, R^2^Y and Q^2^ values were 0.273, 0.981 and 0.964, respectively. The result of permutation test shows that the model is not over-fitted, which verifies the validity of the model (Fig. 1A2,A3).

The corresponding correlation coefficient and VIP values of the metabolite were obtained from the constructed OPLS-DA model, and the fold-change and P value derived from t-test between groups were calculated (Table S1). Volcano map was drawn based on the four parameters, and then potential biomarkers were screened according to the corresponding thresholds (Fig. 1A4). Finally, 19 potential biomarkers of PDAC were screened out. Compared with the control group, the levels of lactate, glutamine, aspartate, leucine, formate, phenylalanine, pyruvate, citrate and isoleucine were higher, while the levels of histidine, β-glucose, low density lipoprotein (LDL), glutamate, valine, trimethylamine, α-glucose, alanine, lipid and very low density lipoprotein (VLDL) were lower in the PDAC group (Table S1).

### The differential metabolites related to the efficacy of chemotherapy in PDAC

Since CA19-9 is an important prognostic indicator of PDAC, the metabolic differences of patients with different CA19-9 levels during chemotherapy were further analyzed in order to find the connection between diagnosis and prognosis of pancreatic cancer in serum metabolome. A total of 24 patients with PDAC were enrolled and divided into two groups according to CA19-9 level. The age and BMI of the PDAC patients followed a normal distribution, and no significant difference was observed between the two stratification according to chi-square test (Table 1).

In order to obtain the distribution profile of all serum samples and determine the internal relationship between the elevated group and the normal group, PCA was performed on the NMR data of all serum samples. The first two principal components provided a cumulative 61.7% explanation rate for the original data, and the two groups showed a separation trend (Fig. 1B1). The subsequent PLS-DA further maximized the systematic variance and removed the disturbing information, and thus the separation between the two groups can be highlighted although moderate overlap is still observed in the score plot (Fig. S2A,B).

In order to clarify the biological significance of CA19-9 in pancreatic cancer patients, OPLS-DA was carried out on the NMR data from the two stratification. The score plots showed that the two groups of samples could be effectively separated with model parameters R_2_X = 0.797, R_2_Y = 0.949, and Q_2_ = 0.60 (Fig. 1B2). The results of permutation test indicated that the model was not overfitted, confirming the validity of the model (Fig. 1B3).

The volcano map was drawn and the results showed that there were significant changes in 14 serum metabolites between the elevated group and the normal group, including the higher levels of citrate, glutamate, isoleucine, arginine, lactate, leucine, VLDL, phosphatidylcholine and LDL, and the lower levels of glutamine, pyruvate, glycerol phosphatidylcholine, alanine and ornithine in the elevated group than in the normal group (Fig. 1B4, Table S2).

### The affected metabolic pathways related to PDAC and chemotherapy

**Figure 2:**
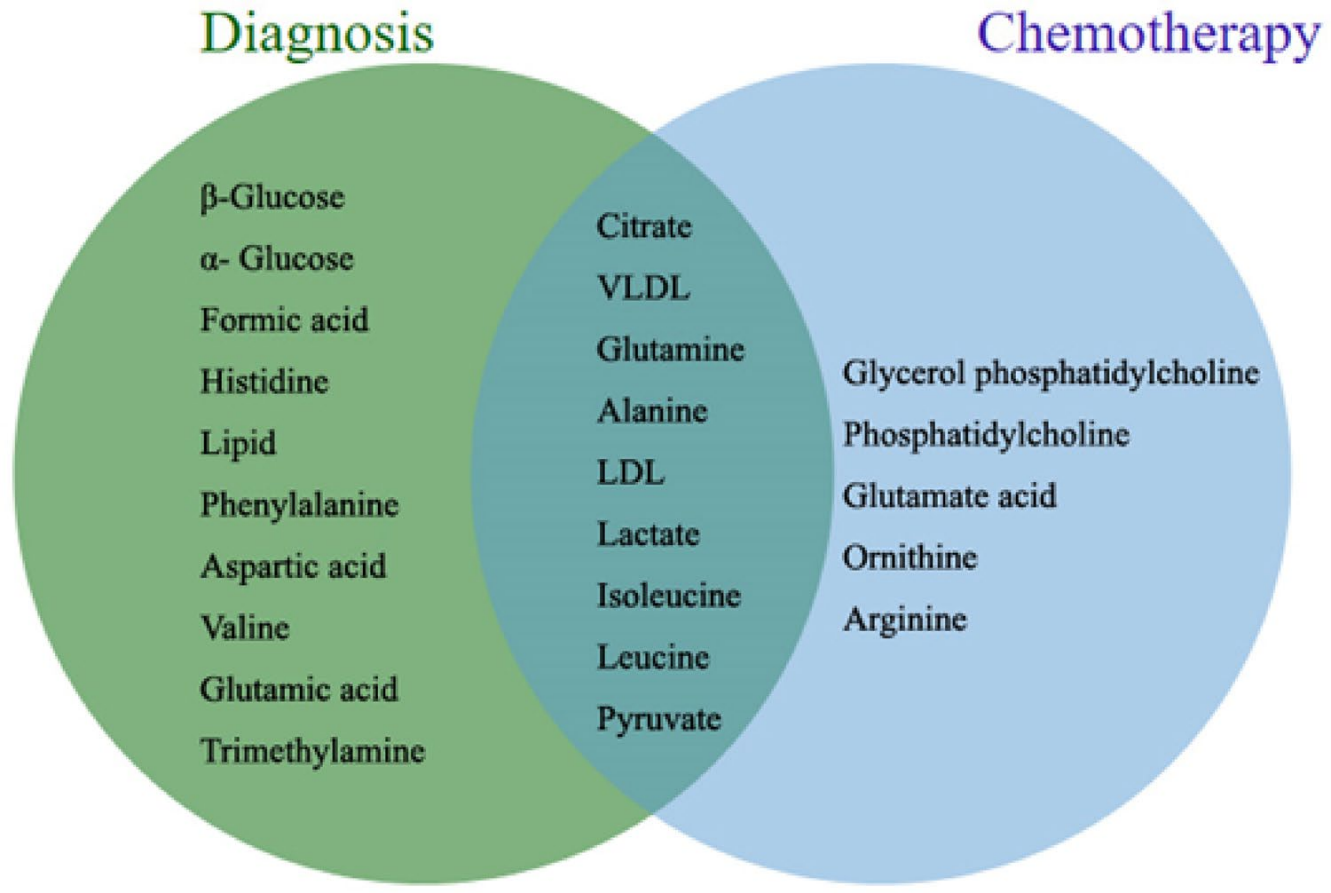
Venn diagram of potential biomarkers for the diagnosis of PDAC and the differential metabolites related to the efficacy of chemotherapy. The intersection represents the presence of similar changes in differential metabolites.

By comparing the potential biomarkers for the diagnosis of PDAC and the differential metabolites related to the efficacy of chemotherapy, it was found that these two set of metabolites were partially overlapped (Fig. 2). Interestingly, the change trend of citrate, alanine, lactate, isoleucine, and leucine in the CA19-9 elevated-normal group was consistent with that in the PDAC vs. control, suggesting that these metabolites might play an important role in the diagnosis and postoperative prognosis of pancreatic cancer.

**Figure 3:**
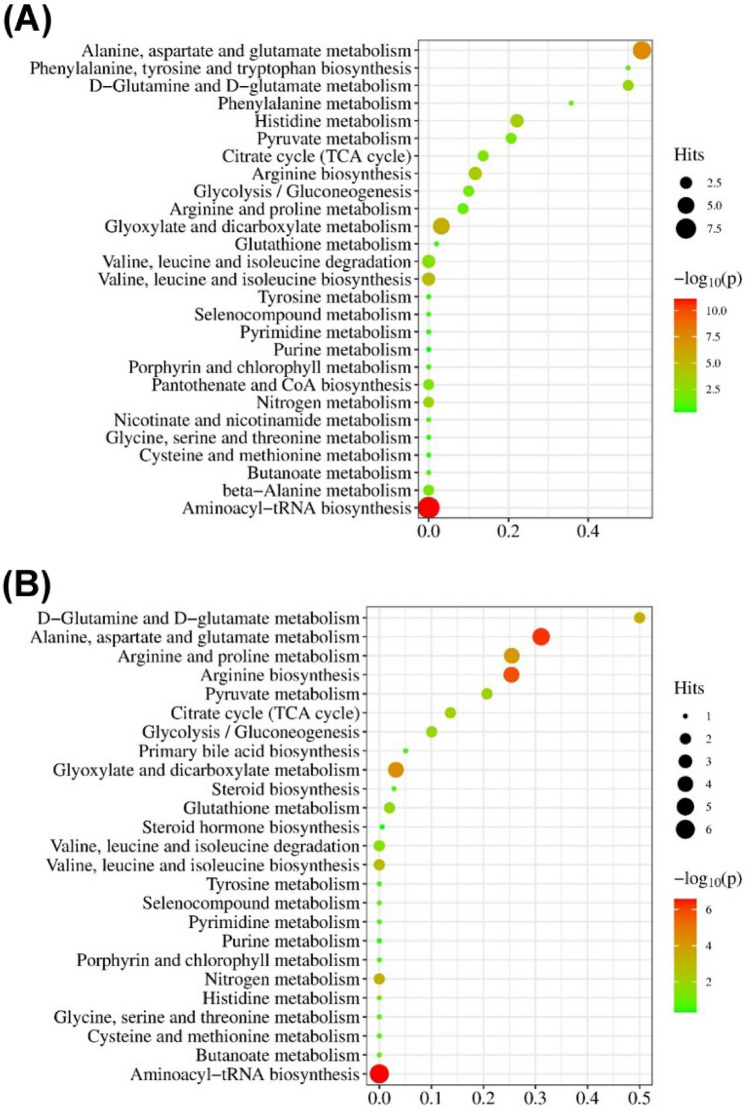
Impact overview of the different metabolic pathways induced by PDAC (**A**) and the CA19-9 level of PDAC patients (**B**).

To determine whether similar metabolic changes are associated with the occurrence of PDAC and poor prognosis, pathway enrichment analysis was conducted on the two sets of differential metabolites. Based on the 19 serum potential biomarkers for PDAC diagnosis, 27 possible metabolic pathways were disturbed as shown in the bubble map of the related pathway (Fig. 3A). Among them, seven metabolic pathways were selected as potential target pathways with a P-value less than 0.05 and a pathway impact greater than 0.1, including alanine, aspartate and glutamate metabolism pathway; arginine biosynthesis pathway; histidine metabolism pathway; D-Glutamine and D-glutamate metabolism pathway; citrate cycle pathway; pyruvate metabolism pathway; and glycolysis/gluconeogenesis pathway (Table 2).

**Table 2:** The target metabolic pathways to PDAC.

Pathway name	Hits	P	− log(P)	Holm P	Impact
Alanine, aspartate and glutamate metabolism	6/28	5.4 × 10^–8^	7.3	4.5 × 10^–6^	0.53
Arginine biosynthesis	3/14	2.0 × 10^–4^	3.7	1.6 × 10^–2^	0.12
Histidine metabolism	3/16	3.1 × 10^–4^	3.5	2.4 × 10^–2^	0.22
d-Glutamine and d-glutamate metabolism	2/6	1.1 × 10^–3^	3.0	8.7 × 10^–2^	0.50
Citrate cycle	2/20	1.3 × 10^–2^	1.9	1.0 × 10^0^	0.14
Pyruvate metabolism	2/22	1.5 × 10^–2^	1.8	1.0 × 10^0^	0.21
glycolysis/gluconeogenesis	2/26	2.2 × 10^–2^	1.7	1.0 × 10^0^	0.10

**Figure 4:**
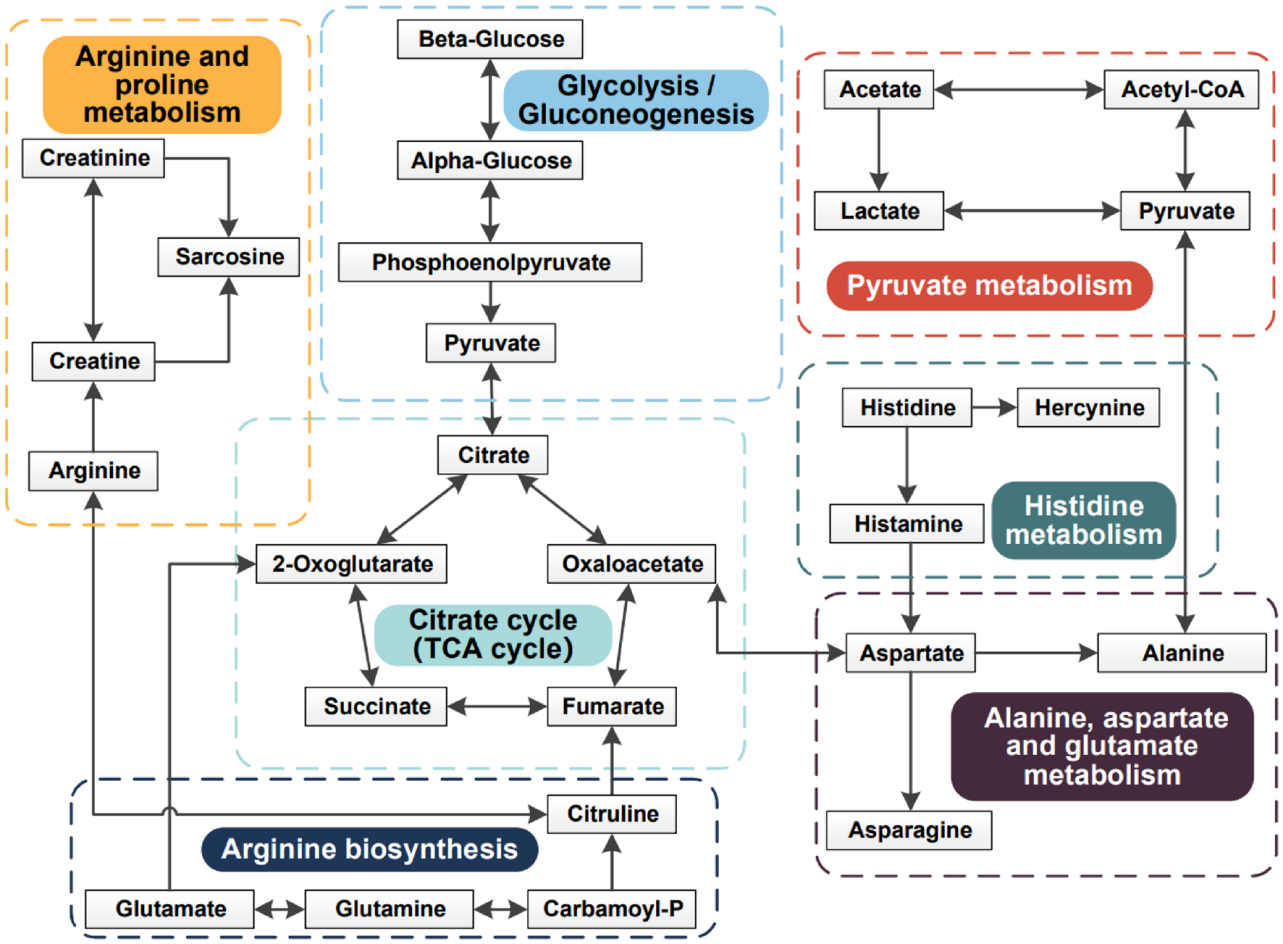
The shared differential metabolic network by PDAC occurrence and CA19-9 level of PDAC patients during chemotherapy.

Similarly, 22 metabolic pathways related to the chemotherapy were obtained by analyzing 14 differential metabolites between the CA19-9 elevated group and normal group (Fig. 3B). Among them, seven pathways with sufficient influence were selected as the important metabolic pathways, including alanine, aspartate and glutamate metabolism pathway; arginine biosynthesis pathway; arginine and proline metabolism pathway; d-Glutamine and d-glutamate metabolism pathway; citrate cycle pathway; pyruvate metabolism pathway; glycolysis/gluconeogenesis pathway (Table 3). By comparing the changes in the two sets of metabolic pathways, six common differential pathways were shared by the occurrence of PDAC and chemotherapy efficacy. These pathways may constitute a metabolic network involved in the pathological development of pancreatic cancer, providing a comprehensive view of metabolic dysregulation (Fig. 4).

**Table 3:** The important metabolic pathways affected by chemotherapy in PDAC.

Pathway name	Hits	P	− log(P)	Holm P	Impact
Alanine, aspartate and glutamate metabolism	5	5.7 × 10^–7^	6.3	4.7 × 10^–5^	0.31
Arginine biosynthesis	4	1.3 × 10^–6^	5.9	1.1 × 10^–4^	0.25
Arginine and proline metabolism	4	9.0 × 10^–5^	4.1	7.2 × 10^–3^	0.25
d-Glutamine and d-glutamate metabolism	2	6.8 × 10^–4^	3.2	5.3 × 10^–2^	0.50
Citrate cycle	2	8.1 × 10^–3^	2.1	6.0 × 10^–1^	0.14
Pyruvate metabolism	2	9.8 × 10^–3^	2.0	7.0 × 10^–1^	0.21
glycolysis/gluconeogenesis	2	1.4 × 10^–2^	1.9	1.0 × 10^0^	0.10

## Discussion

The occurrence of malignant tumors is always accompanied by metabolic changes^16^. The vigorous biological activities of cancer cells and the lack of nutrients in the tumor microenvironment (TME) usually lead to metabolic remodeling of cancer cells to ensure their growth^17^. As a hypoxic tumor, pancreatic cancer shows strong metabolic adaptability in TME. Understanding the metabolic changes during the occurrence and development of pancreatic cancer is helpful to identify the potential biomarkers for the diagnosis and to develop treatment regimen of pancreatic cancer, which can make up for the current dilemma of lack of markers. Metabolomics has shown great application prospects in the discovery of metabolic markers of diseases and has shown great potential in the detection and identification of malignant tumors such as prostate cancer, ovarian cancer, and breast cancer^18–20^. In recent years, some scholars have also screened some metabolic markers related to pancreatic cancer and demonstrated excellent diagnostic efficacy^7^.

In this study, the potential diagnosis biomarkers of PDAC are similar to the differential metabolites of the patients between the elevated and normal CA19-9 groups after chemotherapy. Six metabolic pathways were shared by the occurrence and chemotherapy, mainly focusing on the metabolic changes of glucose, lipids and amino acids.

### Energy metabolism

Warburg effect indicates that the energy supply of normal cells mainly depends on mitochondrial oxidative phosphorylation, while most cancer cells still prefer aerobic glycolysis even if the oxygen supply is sufficient^21^. This relatively low efficiency of energy supply causes different levels of serum glucose, lactate and pyruvate in PDAC patients. Most patients with pancreatic cancer are accompanied by abnormal glucose metabolism, and more than 60% of patients with PDAC have glucose intolerance or diabetes^22^. In addition, higher blood glucose levels are also associated with the risk of pancreatic cancer^23,24^.

In this study, the lactate level was higher in the PDAC group and patients with elevated CA19-9 than their corresponding controls. Lactate, a product of aerobic glycolysis of cells, can stimulate angiogenesis in the TME through vascular endothelial growth factor^25^. TGF-β_2_ can also be upregulated by lactate to promote tumor cell invasion and metastasis^26^. Waldemar et al.^27^ found that lactate can mediate the acetylation of histone H3 and H4, up-regulate DNA repair genes, and improve the survival ability of tumor cells after chemotherapy. In addition, lactate could inhibit the production of IFN-γ and IL-4 in the TME and induce NK cell dysfunction^28^.

### Lipid metabolism

Lipids can provide energy for tumor cell proliferation and raw materials for membrane synthesis^29^. Therefore, the occurrence and development of PDAC cannot be separated from the changes in lipid metabolism. This study found changes in lipid metabolism in PDAC patients and chemotherapy groups. Serum lipoproteins were also associated with pancreatic cancer. The study of patients with malignant tumors found the changed levels of serum high-density lipoprotein and apolipoprotein A-I^30^. By analyzing the metabolic profile, some studies have found that the proliferation of PDAC cells is related to the level of plasma LDL^31^.

Based on the MALDI-TOF–MS platform, metabolic disorders of phosphatidylcholine have been found to have important application value in monitoring the progression and generation of drug resistance after targeted therapy in patients with cancer^32^. Therefore, monitoring the changes of serum phosphatidylcholine levels may have predictive value for the prognosis of PDAC. The main function of VLDL is to transport endogenous triglycerides into peripheral blood, and most of them are converted into LDL. In vitro experiments found that silencing the low density lipoprotein receptor (LDLR) with shRNA can significantly reduce the intake of cholesterol, inhibit the progression of PDAC by blocking the ERK1/2 pathway, and improve the sensitivity of chemotherapy^33^. Similarly, gemcitabine resistance can be reduced by blocking ERK/JNK pathway^34^. Therefore, it is speculated that LDL can activate the ERK1/2 signaling pathway by binding to LDLR to promote progression and resistance to chemotherapy, leading to a poor prognosis.

### Amino acid metabolism

In this study, it was found that the levels of branched-chain amino acids (BCAA), glutamine, arginine and other amino acids changed in patients with PDAC occurrence and during postoperative chemotherapy. It suggests that amino acid metabolic remodeling occurs during the proliferation of PDAC.

When the tumor is in a hypoglycemic state, amino acids and fatty acids (FAA) will be used for energy supply through the citric acid cycle^35^. As the most abundant amino acid in plasma, glutamine is often used as an alternative energy source. Alanine is converted into glutamate through transaminase, and then glutamine is synthesized. In normal cells, glutamine is rarely used to provide energy, whereas tumor cells induce metabolic reprogramming of glutamine via Kras and MYC, leading to glutamine addiction in cancer^36,37^. In tumor cells, glutamine is hydrolyzed into glutamate, which enters the mitochondria and participates in the citric acid cycle, becoming an energy source for cancer cell growth. Glutamine provides a source of carbon and nitrogen for tumor biosynthesis and energy metabolism. Meanwhile, as an important reducing agent in vivo, glutamine also plays a non-negligible role in maintaining cellular homeostasis^38^. Biancur et al.^39^ found that the use of glutaminase inhibitors can inhibit the proliferation and metastasis of pancreatic cancer cells. Wang et al.^40^ found that CD-9 enhanced glutamine uptake and proliferation of cancer cells by interacting with glutamine transporters in a PANC-1 xenograft mouse model. More interestingly, by interfering with glutamine metabolism, the drug sensitivity of gemcitabine-resistant pancreatic cancer cell lines increased^41^. Therefore, as an energy source of pancreatic cancer cells, the change of serum glutamine level may predict the progression of the disease.

Arginine can be metabolized to ornithine and participate in the ornithine cycle. More than 80% of pancreatic cancer is arginine auxotrophy, which means that most pancreatic cancer cells depend on exogenous arginine for life activities^42^. Arginine and its metabolites play an important role in the genesis and progression of pancreatic cancer cells, and arginine deprivation therapy is becoming a research hot spot^43^. Studies have found that hydrolyzing arginine with arginine deiminase can induce pancreatic cancer cells to stay in the S phase and up-regulate the expressions of caspase-3 and caspase-9. In combination with gemcitabine, better antitumor activity could be obtained^44^.

Leucine, isoleucine and valine are collectively known as BCAA. It is believed that both BCAA and glutamine can provide carbon and nitrogen sources for cancer cell biosynthesis and promote tumor growth^45–47^. In recent years, many studies have found the varied levels of glutamine and BCAA in pancreatic cancer patients^48,49^. In somatic cells, BCAA participates in the citric acid cycle by transamination and decarboxylation to generate fatty acyl-CoA. At present, the metabolic remodeling of BCAA has received extensive attention in the occurrence and development of pancreatic cancer. Research has found that elevated plasma BCAA levels are associated with the future risk of pancreatic cancer.

BCAA can provide energy for the growth of cancer cells by supporting mitochondrial biosynthesis and inducing autophagy, which confirms the high level of BCAA in pancreatic cancer patients^50,51^. Similar to glutamine, BCAA can also provide carbon and nitrogen sources for tumors. Lee et al.^46^ found that PDAC played an important role in the growth of PDAC by using BCAAs as carbon sources for FAA biosynthesis. Knockout of BCAT 2 or BCAAD significantly reduced the proliferation of PDAC cells and inhibited the synthesis of FAA. Similarly, the study of pancreatic tissue of KC mice found that BCAA also provided nitrogen source for the synthesis of non-essential amino acids^47^. The increase in BCAA levels in patients with elevated CA19-9 suggests that the metabolic reprogramming of BCAA is also related to the progression of cancer. Ericksen et al.^52^ combined transcriptomics, enzymology and metabolomics to compare cancer and paracancerous tissue samples, and found that the defects of key enzymes in the process of BCAA metabolism led to the excessive accumulation of BCAA in cells, thus promoting tumor progression by enhancing the activity of mTORC1. Coincidentally, a targeted metabolomic analysis of 16 amino acids in the plasma of patients with myeloid leukemia found that the level of BCAA in the blastic phase was higher than that in the chronic phase^53^.

### Citric acid metabolism

The glycolysis product pyruvate is oxidized and decarboxylated to produce acetyl-CoA, which then reacts with oxaloacetic acid to synthesize citric acid. Citric acid generates oxaloacetic acid through a series of reactions, thus forming citric acid cycle. Acetyl-CoA is not only the raw material of FAA synthesis but also the product of FAA oxidation. Glutamate, valine, phenylalanine, aspartate, and leucine also participate in the synthesis of intermediate products of the citric acid cycle through a variety of ways. Therefore, citric acid cycle is the hub of glucose, lipid and amino acid metabolism, and plays a central role in the metabolism of cells^54^. Elevated citrate levels were observed in patients with PDAC and those with elevated CA19-9, indicating changes in citric acid metabolism.

Previous evidence has shown that cancer related fibroblasts can produce citrate to support tumor progression and enhance drug resistance of cancer cells, and removing citrate can significantly inhibit cancer cell growth and metastasis, as well as epithelial-mesenchymal transformation and angiogenesis^55,56^, implying the possible treatment regimen of PDAC by decreasing the citrate levels.

In this study, 19 and 14 differential metabolites were screened out from the two sets of comparisons. The analysis found that the differential metabolites screened from the two cohorts were highly correlated, including lactate, pyruvate, glutamate, glutamine, BCAA, citrate and other differential metabolites with similar change trends. Further pathway analysis of differential metabolites revealed that there were 6 identical metabolic pathways in both groups of experiments. These results suggest that similar metabolic differences accompany the occurrence and development of pancreatic cancer. The selection of appropriate metabolic markers or combinations of markers will provide effective help for the diagnosis and prognosis evaluation of pancreatic cancer.

However, there are some limitations in our study. The study only included PDAC patients with elevated preoperative CA19-9 level. But in clinical practice, some patients are Lewis antigen negative and present no elevated CA19-9 levels. Whether the conclusion in this study could extend to these patients keeps unknown. More importantly, the number of samples in this study is small, and the reliability of the conclusion needs to be confirmed by a multi-center study with a larger sample size.

## Conclusion

NMR-based metabolomics can effectively distinguish the metabolic characteristics of PDAC patients from the controls and between those with normal and elevated CA19-9 levels during postoperative chemotherapy. There are significant differences in energy metabolism, lipid metabolism, amino acid metabolism, and citric acid metabolism between the two CA19-9 stratification. The similarity between the metabolic characteristics induced by PDAC and the metabolic responses to chemotherapy indirectly suggests that some metabolic reactions may be involved in the entire process of pancreatic cancer development. Therefore, the selection of appropriate differential metabolites or metabolite combinations can provide a reference for early clinical diagnosis and prognosis prediction of pancreatic cancer patients.

### Supplementary Information

Supplementary Information.

## Data Availability

The data involved in this article are available at the Metabolomics Workbench (https://www.metabolomicsworkbench.org). Further information and other data that support the findings of this study are available from the corresponding author upon reasonable request.

## Abbreviations

BCAA: Branched-chain amino acids
BMI: Body mass index
CA19-9: Carbohydrate antigen 19-9
CPMG: Carr–Purcell–Meiboom–Gill
KHz: Kilohertz
FAA: Fatty acids
LDL: Low density lipoprotein
LDLR: Low density lipoprotein receptor
MHz: Megahertz
NMR: Nuclear magnetic resonance
OPLS-DA: Orthogonal partial least squares-discrimination analysis
PCA: Principal component analysis
PDAC: Pancreatic ductal adenocarcinoma
PLS-DA: Partial least squares-discrimination analysis
ROC: Receiver operating characteristic
TME: Tumor microenvironment
TNM: Tumor node metastasis
VIP: Variable importance for the projection
VLDL: Very low density lipoprotein
