# Supplementary material for: Metabolic responses to the occurrence and chemotherapy of pancreatic cancer: biomarker identification and prognosis prediction
Source: Sci Rep. 2024 Mar 23;14:6938. doi: 10.1038/s41598-024-56737-4 (PMC10960848; doi:10.1038/s41598-024-56737-4)
Supplement: Supplementary file 1 — Supplementary Information. [file 41598_2024_56737_MOESM1_ESM.pdf]

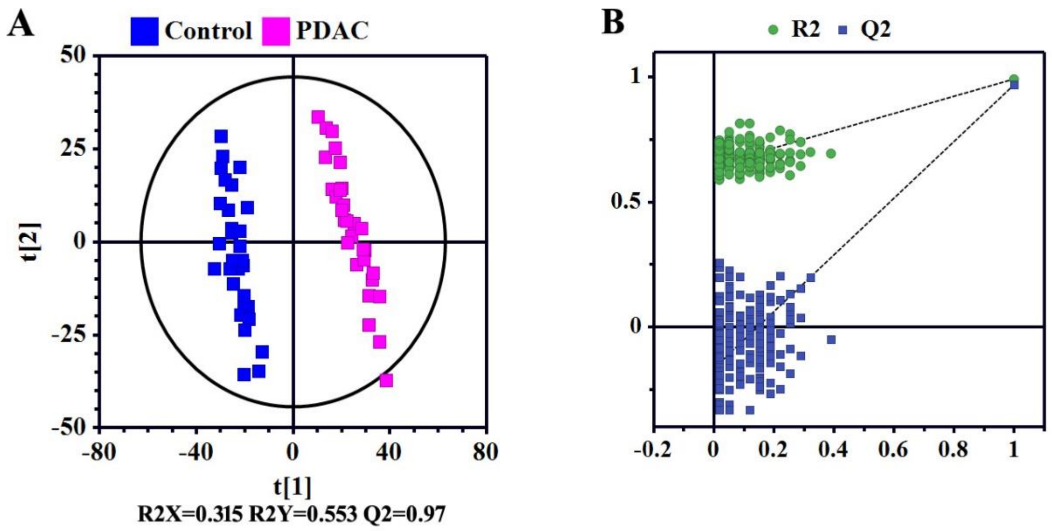

Fig.S1. PLS-DA score plots (A) and the corresponding permutation test (n=200) (B) from the PDAC patients and the healthy controls.

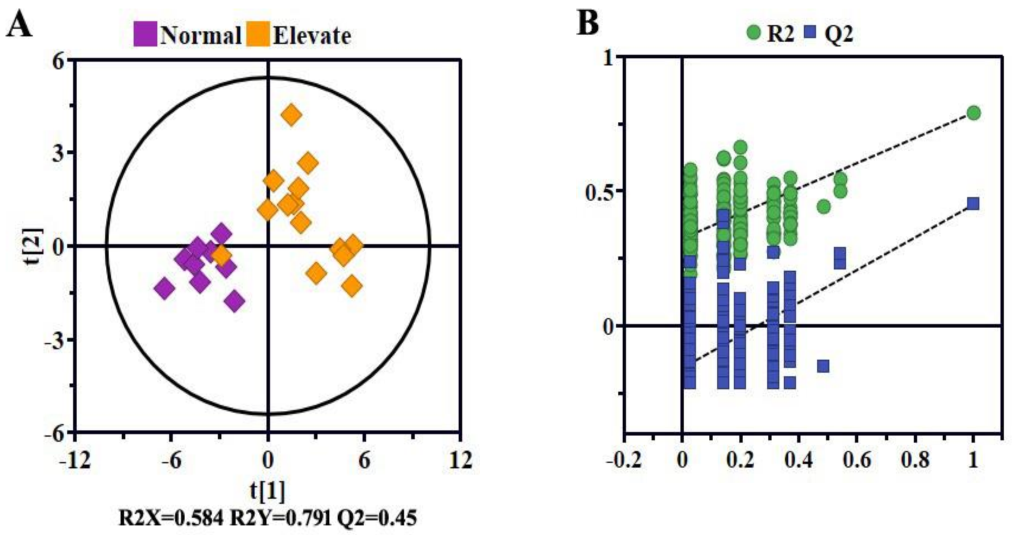

Fig.S2. PLS-DA score plots (A) and the corresponding permutation test (n=200) (B) from two stratification of PDAC patients.

Table S1. The potential biomarkers of PDAC

Potential biomarkers	r	P value	FoldChange	VIP[a]	Change (PDAC group vs. control group)
Lactate	0.937	2.9×10^{-6}	0.432	1.416	↑
Glutamine	0.796	2.8×10^{-4}	0.717	1.759	↑
Histidine	-0.817	8.0×10^{-4}	1.416	1.454	↓
β-Glucose	-0.944	9.7×10^{-4}	1.747	1.761	↓
Aspartate	0.773	1.9×10^{-3}	0.443	1.698	↑
Leucine	0.708	2.0×10^{-3}	0.620	1.756	↑
LDL	-0.776	4.0×10^{-3}	1.330	1.355	↓
Formate	0.706	4.0×10^{-3}	0.221	1.085	↑
Glutamate	-0.762	4.2×10^{-3}	1.374	1.209	↓
Valine	-0.752	4.6×10^{-3}	2.483	1.627	↓
Phenylalanine	0.707	5.6×10^{-3}	0.167	2.027	↑
Trimethylamine	-0.746	7.8×10^{-3}	1.387	1.575	↓
α-Glucose	-0.701	8.3×10^{-3}	1.170	1.323	↓
Pyruvate	0.731	9.5×10^{-3}	0.300	1.668	↑
Citrate	0.754	3.0×10^{-2}	0.744	1.217	↑
Alanine	-0.724	4.1×10^{-2}	1.318	1.396	↓
Lipid	-0.759	4.2×10^{-2}	1.962	1.666	↓
VLDL	-0.740	4.7×10^{-2}	1.419	1.391	↓
Isoleucine	0.745	4.9×10^{-2}	0.534	1.524	↑

"↑" or "↓" indicates that the levels of metabolites in the PDAC group are higher or lower than that in the control group. The potential biomarkers must simultaneously meet the requirements of VIP >1, r >0.7 and p <0.05. [a]Variable importance in projection.

Table S2. Differential metabolites between the normal group and elevated group

Differential metabolites	r	P value	FoldChange	VIP	Change (elevated group vs. normal group）
Citrate	0.899	2.4×10^{-4}	0.672	2.022	↑
Glutamate	0.755	5.6×10^{-4}	0.483	1.402	↑
Isoleucine	0.749	8.5×10^{-4}	0.753	1.251	↑
Glutamine	-0.664	2.8×10^{-3}	2.078	2.020	↓
Pyruvate	-0.600	3.6×10^{-3}	1.624	1.048	↓
Arginine	0.738	5.2×10^{-3}	0.379	1.566	↑
Lactate	0.659	6.6×10^{-3}	0.656	1.648	↑
Leucine	0.599	9.6×10^{-3}	0.897	1.112	↑
Glycerol phosphatidylcholine	-0.697	1.0×10^{-2}	1.826	1.551	↓
VLDL	0.576	1.6×10^{-2}	0.525	1.250	↑
Alanine	-0.578	1.9×10^{-2}	1.853	2.054	↓
Phosphatidylcholine	0.670	2.8×10^{-2}	0.798	1.941	↑
Ornithine	-0.566	4.2×10^{-2}	1.329	1.211	↓
LDL	0.721	4.8×10^{-2}	0.874	1.360	↑

"↑" and "↓"indicates that the levels of metabolites in the normal group are higher and lower than that in the elevated group, respectively.
